# 2-[(3*R*,6*R*)-6-Methyl-2,5-dioxomorph­olin-3-yl]-*N*-(propan-2-yl)acetamide

**DOI:** 10.1107/S1600536812007945

**Published:** 2012-02-29

**Authors:** De-dai Lu, Hu Zhang, Juan Luo, Li-qiang Yang, Peng-xue Duan

**Affiliations:** a Key Laboratory of Polymer Materials of Gansu Province, Key Laboratory of Eco-Environment-Related Polymer Materials, Ministry of Education, College of Chemistry and Chemical Engineering, Northwest Normal University, Lanzhou 730070, People’s Republic of China

## Abstract

The molecular conformation of the title compound, C_10_H_16_N_2_O_4_, is determined by an intra­molecular N—H⋯O hydrogen bond involving the morpholine NH group and the amide O atom. In the crystal, mol­ecules are linked by N—H⋯O hydrogen bonds into chains along the *a*-axis direction.

## Related literature

For the synthesis of polydepsipeptides, see: Feng *et al.* (2002 ▶); Hughes & Sleebs (2005 ▶); In’t Veld *et al.* (1992 ▶,1994 ▶); Jörres *et al.* (1998 ▶). For the synthesis of title compound, see: Wang & Feng (1997 ▶).
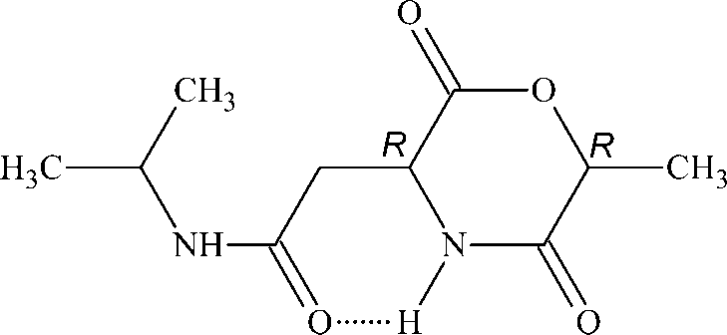


## Experimental

### 

#### Crystal data

C_10_H_16_N_2_O_4_
*M* *_r_* = 228.25
Monoclinic, 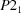

*a* = 8.038 (3) Å
*b* = 5.678 (2) Å
*c* = 12.656 (5) Å
β = 105.476 (4)°
*V* = 556.6 (3) Å^3^
*Z* = 2
Mo *K*α radiation
μ = 0.11 mm^−1^
*T* = 296 K
0.28 × 0.26 × 0.24 mm

#### Data collection

Bruker APEXII CCD diffractometer
Absorption correction: multi-scan (*SADABS*; Bruker, 2009 ▶) *T*_min_ = 0.971, *T*_max_ = 0.975
3796 measured reflections
1111 independent reflections
973 reflections with *I* > 2σ(*I*)
*R*_int_ = 0.026

#### Refinement

*R*[*F* ^2^ > 2σ(*F* ^2^)] = 0.036
*wR*(*F* ^2^) = 0.085
*S* = 1.16
1111 reflections
153 parameters
1 restraint
H atoms treated by a mixture of independent and constrained refinement
Δρ_max_ = 0.13 e Å^−3^
Δρ_min_ = −0.13 e Å^−3^

### 

Data collection: *APEX2* (Bruker, 2009 ▶); cell refinement: *SAINT* (Bruker, 2009 ▶); data reduction: *SAINT*; program(s) used to solve structure: *SHELXS97* (Sheldrick, 2008 ▶); program(s) used to refine structure: *SHELXL97* (Sheldrick, 2008 ▶); molecular graphics: *SHELXTL* (Sheldrick, 2008 ▶); software used to prepare material for publication: *SHELXTL*.

## Supplementary Material

Crystal structure: contains datablock(s) I, global. DOI: 10.1107/S1600536812007945/yk2036sup1.cif

Structure factors: contains datablock(s) I. DOI: 10.1107/S1600536812007945/yk2036Isup2.hkl

Supplementary material file. DOI: 10.1107/S1600536812007945/yk2036Isup3.cml

Additional supplementary materials:  crystallographic information; 3D view; checkCIF report

## Figures and Tables

**Table 1:** Hydrogen-bond geometry (Å, °)

*D*—H⋯*A*	*D*—H	H⋯*A*	*D*⋯*A*	*D*—H⋯*A*
N1—H1N⋯O4	0.85 (4)	2.09 (3)	2.763 (4)	136 (3)
N2—H2N⋯O1^i^	0.82 (3)	2.11 (3)	2.926 (3)	170 (3)

Symmetry code: (i) 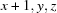.

**Fig. 1.**
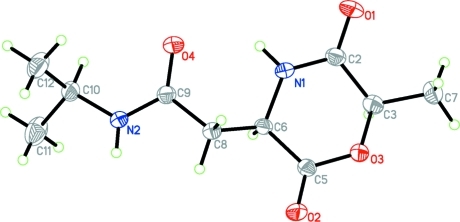
Molecular structure of the title compound showing the atom labelling scheme. Displacement ellipsoids are drawn at the 30% probability level.

**Fig. 2.**
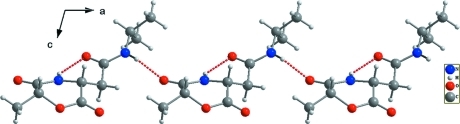
The hydrogen-bonded chain in the structure of title compound.

## supplementary crystallographic information

### Comment

Polydepsipeptides, copolymers of α-hydroxy acids and α-amino acids, are the most important representatives of biodegradable polyesteramides. Morpholine-2,5-dione derivatives are a series of monomers which were used to synthesize polydepsipeptides *via* ring-opening polymerization. (In't Veld *et al.*, 1992,1994). In recent years, all kinds of functional groups are introduced into these monomers in order to synthesize functional polydepsipeptides (Feng *et al.*, 2002; Jörres *et al.*, 1998; Hughes & Sleebs, 2005). In our current research, related to this topic, we have designed and synthesized the title compound, which includes the isopropyl amide functional group (Fig. 1). In the crystal structure of the title compound, C_10_H_16_N_2_O_4_, there are two kinds of hydrogen bonds, one is intramolecular N—H···O [H···O = 2.09 (3) Å] hydrogen bond, the other is intermolecular N—H···O [H···O = 2.11 (3) Å] hydrogen bond which links molecules into the one-dimensional chains along the *a*-axis direction (Fig. 2).

### Experimental

All reagents used in the syntheses were of analytical grade and used without further purification. The title compound was prepared according to the literature method (Wang & Feng, 1997). Single crystals were grown from ethyl acetate solution by slow evaporation at room temperature.

### Refinement

All H atoms were placed in calculated positions, with C—H distances ranging from 0.96 to 0.98 Å, and N—H distances ranging from 0.82 to 0.85 Å. They were refined in the riding-model approximation, with *U*_iso_(H) = 1.2*U*_eq_(carrier C, N) or *U*_iso_(H) = 1.5*U*_eq_(C of methyl group).

### Crystal data

**Table:**

C_10_H_16_N_2_O_4_	*F*(000) = 244
*M**_r_* = 228.25	*D*_x_ = 1.362 Mg m^−^^3^
Monoclinic, *P*2_1_	Mo *K*α radiation, λ = 0.71073 Å
Hall symbol: P 2yb	Cell parameters from 2019 reflections
*a* = 8.038 (3) Å	θ = 2.6–28.4°
*b* = 5.678 (2) Å	µ = 0.11 mm^−^^1^
*c* = 12.656 (5) Å	*T* = 296 K
β = 105.476 (4)°	Block, colourless
*V* = 556.6 (3) Å^3^	0.28 × 0.26 × 0.24 mm
*Z* = 2	

### Data collection

**Table:**

Bruker APEXII CCD diffractometer	1111 independent reflections
Radiation source: fine-focus sealed tube	973 reflections with *I* > 2σ(*I*)
Graphite monochromator	*R*_int_ = 0.026
φ and ω scans	θ_max_ = 25.5°, θ_min_ = 2.6°
Absorption correction: multi-scan (*SADABS*; Bruker, 2009)	*h* = −9→8
*T*_min_ = 0.971, *T*_max_ = 0.975	*k* = −6→6
3796 measured reflections	*l* = −15→15

### Refinement

**Table:**

Refinement on *F*^2^	Primary atom site location: structure-invariant direct methods
Least-squares matrix: full	Secondary atom site location: difference Fourier map
*R*[*F*^2^ > 2σ(*F*^2^)] = 0.036	Hydrogen site location: inferred from neighbouring sites
*wR*(*F*^2^) = 0.085	H atoms treated by a mixture of independent and constrained refinement
*S* = 1.16	*w* = 1/[σ^2^(*F*_o_^2^) + (0.0381*P*)^2^ + 0.0663*P*] where *P* = (*F*_o_^2^ + 2*F*_c_^2^)/3
1111 reflections	(Δ/σ)_max_ < 0.001
153 parameters	Δρ_max_ = 0.13 e Å^−^^3^
1 restraint	Δρ_min_ = −0.13 e Å^−^^3^

### Special details

**Table:**

Geometry. All s.u.'s (except the s.u. in the dihedral angle between two l.s. planes) are estimated using the full covariance matrix. The cell s.u.'s are taken into account individually in the estimation of s.u.'s in distances, angles and torsion angles; correlations between s.u.'s in cell parameters are only used when they are defined by crystal symmetry. An approximate (isotropic) treatment of cell s.u.'s is used for estimating s.u.'s involving l.s. planes.
Refinement. Refinement of *F*^2^ against ALL reflections. The weighted *R*-factor *wR* and goodness of fit *S* are based on *F*^2^, conventional *R*-factors *R* are based on *F*, with *F* set to zero for negative *F*^2^. The threshold expression of *F*^2^ > σ(*F*^2^) is used only for calculating *R*-factors(gt) *etc*. and is not relevant to the choice of reflections for refinement. *R*-factors based on *F*^2^ are statistically about twice as large as those based on *F*, and *R*- factors based on ALL data will be even larger.

### Fractional atomic coordinates and isotropic or equivalent isotropic displacement parameters (Å^2^)

**Table:**

	*x*	*y*	*z*	*U*_iso_*/*U*_eq_	
N1	0.3090 (3)	0.0143 (5)	0.7877 (2)	0.0396 (6)	
H1N	0.303 (3)	−0.121 (7)	0.759 (2)	0.041 (9)*	
O1	0.0310 (2)	0.0204 (5)	0.78753 (18)	0.0587 (6)	
C2	0.1717 (3)	0.1165 (6)	0.8051 (2)	0.0395 (7)	
N2	0.7140 (3)	−0.2115 (4)	0.66322 (19)	0.0384 (6)	
H2N	0.803 (4)	−0.138 (6)	0.691 (2)	0.041 (9)*	
O2	0.6493 (2)	0.3811 (4)	0.93137 (16)	0.0480 (6)	
C3	0.1981 (3)	0.3664 (6)	0.8450 (2)	0.0425 (7)	
H3A	0.1803	0.4699	0.7810	0.051*	
O3	0.3725 (2)	0.4038 (4)	0.91393 (15)	0.0426 (5)	
O4	0.4538 (2)	−0.3182 (4)	0.67977 (16)	0.0500 (6)	
C5	0.5079 (3)	0.3148 (5)	0.8859 (2)	0.0343 (7)	
C6	0.4708 (3)	0.1354 (5)	0.7952 (2)	0.0310 (6)	
H6A	0.4607	0.2184	0.7259	0.037*	
C7	0.0782 (4)	0.4419 (7)	0.9102 (3)	0.0641 (11)	
H7A	0.1023	0.6023	0.9332	0.096*	
H7B	0.0939	0.3426	0.9735	0.096*	
H7C	−0.0388	0.4295	0.8659	0.096*	
C8	0.6152 (3)	−0.0437 (5)	0.8098 (2)	0.0351 (7)	
H8A	0.7244	0.0380	0.8203	0.042*	
H8B	0.6210	−0.1370	0.8749	0.042*	
C9	0.5878 (3)	−0.2038 (5)	0.7122 (2)	0.0355 (7)	
C10	0.7090 (3)	−0.3549 (6)	0.5672 (2)	0.0409 (7)	
H10A	0.5898	−0.3578	0.5211	0.049*	
C11	0.8204 (5)	−0.2421 (7)	0.5025 (3)	0.0704 (11)	
H11A	0.7822	−0.0836	0.4839	0.106*	
H11B	0.8118	−0.3306	0.4366	0.106*	
H11C	0.9384	−0.2403	0.5458	0.106*	
C12	0.7629 (5)	−0.6010 (7)	0.5983 (3)	0.0634 (10)	
H12A	0.6895	−0.6672	0.6392	0.095*	
H12B	0.8803	−0.6023	0.6424	0.095*	
H12C	0.7538	−0.6927	0.5332	0.095*	

### Atomic displacement parameters (Å^2^)

**Table:**

	*U*^11^	*U*^22^	*U*^33^	*U*^12^	*U*^13^	*U*^23^
N1	0.0359 (13)	0.0329 (15)	0.0509 (16)	−0.0098 (12)	0.0133 (11)	−0.0121 (14)
O1	0.0304 (11)	0.0629 (16)	0.0810 (15)	−0.0155 (11)	0.0121 (10)	−0.0133 (14)
C2	0.0326 (15)	0.0445 (19)	0.0402 (15)	−0.0056 (14)	0.0077 (12)	−0.0024 (14)
N2	0.0345 (13)	0.0397 (15)	0.0424 (14)	−0.0085 (12)	0.0127 (11)	−0.0152 (12)
O2	0.0342 (10)	0.0475 (13)	0.0588 (12)	−0.0081 (11)	0.0064 (9)	−0.0169 (11)
C3	0.0330 (14)	0.0476 (19)	0.0453 (17)	−0.0025 (15)	0.0073 (12)	−0.0029 (15)
O3	0.0322 (9)	0.0479 (13)	0.0481 (11)	−0.0059 (10)	0.0115 (8)	−0.0154 (10)
O4	0.0389 (11)	0.0542 (15)	0.0574 (13)	−0.0152 (11)	0.0138 (9)	−0.0229 (12)
C5	0.0346 (15)	0.0343 (18)	0.0359 (14)	−0.0045 (13)	0.0125 (11)	0.0009 (13)
C6	0.0299 (13)	0.0330 (16)	0.0305 (14)	−0.0062 (12)	0.0088 (10)	−0.0015 (13)
C7	0.0410 (17)	0.077 (3)	0.077 (2)	−0.0029 (19)	0.0215 (15)	−0.028 (2)
C8	0.0330 (14)	0.0361 (18)	0.0351 (14)	−0.0045 (13)	0.0072 (11)	−0.0050 (13)
C9	0.0340 (14)	0.0340 (17)	0.0375 (15)	−0.0004 (13)	0.0077 (11)	−0.0011 (14)
C10	0.0370 (15)	0.0457 (19)	0.0394 (16)	0.0007 (15)	0.0091 (12)	−0.0102 (15)
C11	0.112 (3)	0.051 (2)	0.063 (2)	−0.001 (2)	0.050 (2)	−0.003 (2)
C12	0.091 (3)	0.039 (2)	0.069 (2)	0.002 (2)	0.035 (2)	−0.0075 (19)

### Geometric parameters (Å, º)

**Table:**

N1—C2	1.317 (4)	C6—H6A	0.9800
N1—C6	1.452 (3)	C7—H7A	0.9600
N1—H1N	0.85 (4)	C7—H7B	0.9600
O1—C2	1.222 (3)	C7—H7C	0.9600
C2—C3	1.502 (5)	C8—C9	1.502 (4)
N2—C9	1.323 (3)	C8—H8A	0.9700
N2—C10	1.454 (4)	C8—H8B	0.9700
N2—H2N	0.82 (3)	C10—C12	1.485 (5)
O2—C5	1.190 (3)	C10—C11	1.508 (4)
C3—O3	1.456 (3)	C10—H10A	0.9800
C3—C7	1.489 (4)	C11—H11A	0.9600
C3—H3A	0.9800	C11—H11B	0.9600
O3—C5	1.331 (3)	C11—H11C	0.9600
O4—C9	1.231 (3)	C12—H12A	0.9600
C5—C6	1.504 (4)	C12—H12B	0.9600
C6—C8	1.517 (4)	C12—H12C	0.9600
			
C2—N1—C6	123.8 (3)	H7A—C7—H7C	109.5
C2—N1—H1N	121.1 (19)	H7B—C7—H7C	109.5
C6—N1—H1N	114.0 (19)	C9—C8—C6	111.5 (2)
O1—C2—N1	123.3 (3)	C9—C8—H8A	109.3
O1—C2—C3	121.6 (3)	C6—C8—H8A	109.3
N1—C2—C3	115.0 (3)	C9—C8—H8B	109.3
C9—N2—C10	123.8 (3)	C6—C8—H8B	109.3
C9—N2—H2N	118 (2)	H8A—C8—H8B	108.0
C10—N2—H2N	118 (2)	O4—C9—N2	122.6 (3)
O3—C3—C7	106.7 (2)	O4—C9—C8	121.1 (2)
O3—C3—C2	111.5 (3)	N2—C9—C8	116.3 (2)
C7—C3—C2	113.8 (3)	N2—C10—C12	111.4 (3)
O3—C3—H3A	108.2	N2—C10—C11	109.1 (3)
C7—C3—H3A	108.2	C12—C10—C11	111.7 (3)
C2—C3—H3A	108.2	N2—C10—H10A	108.2
C5—O3—C3	120.7 (2)	C12—C10—H10A	108.2
O2—C5—O3	119.6 (3)	C11—C10—H10A	108.2
O2—C5—C6	123.5 (2)	C10—C11—H11A	109.5
O3—C5—C6	116.8 (2)	C10—C11—H11B	109.5
N1—C6—C5	111.3 (2)	H11A—C11—H11B	109.5
N1—C6—C8	109.5 (2)	C10—C11—H11C	109.5
C5—C6—C8	111.9 (2)	H11A—C11—H11C	109.5
N1—C6—H6A	108.0	H11B—C11—H11C	109.5
C5—C6—H6A	108.0	C10—C12—H12A	109.5
C8—C6—H6A	108.0	C10—C12—H12B	109.5
C3—C7—H7A	109.5	H12A—C12—H12B	109.5
C3—C7—H7B	109.5	C10—C12—H12C	109.5
H7A—C7—H7B	109.5	H12A—C12—H12C	109.5
C3—C7—H7C	109.5	H12B—C12—H12C	109.5
			
C6—N1—C2—O1	172.1 (3)	O2—C5—C6—N1	155.1 (3)
C6—N1—C2—C3	−5.8 (4)	O3—C5—C6—N1	−25.9 (3)
O1—C2—C3—O3	149.0 (3)	O2—C5—C6—C8	32.4 (4)
N1—C2—C3—O3	−33.1 (3)	O3—C5—C6—C8	−148.7 (2)
O1—C2—C3—C7	28.2 (4)	N1—C6—C8—C9	63.2 (3)
N1—C2—C3—C7	−153.9 (3)	C5—C6—C8—C9	−173.0 (2)
C7—C3—O3—C5	168.1 (3)	C10—N2—C9—O4	−0.3 (4)
C2—C3—O3—C5	43.3 (4)	C10—N2—C9—C8	−179.4 (3)
C3—O3—C5—O2	166.4 (3)	C6—C8—C9—O4	−55.6 (3)
C3—O3—C5—C6	−12.6 (4)	C6—C8—C9—N2	123.5 (3)
C2—N1—C6—C5	36.3 (4)	C9—N2—C10—C12	−83.3 (3)
C2—N1—C6—C8	160.4 (2)	C9—N2—C10—C11	152.9 (3)

### Hydrogen-bond geometry (Å, º)

**Table:**

*D*—H···*A*	*D*—H	H···*A*	*D*···*A*	*D*—H···*A*
N1—H1*N*···O4	0.85 (4)	2.09 (3)	2.763 (4)	136 (3)
N2—H2*N*···O1^i^	0.82 (3)	2.11 (3)	2.926 (3)	170 (3)

Symmetry code: (i) *x*+1, *y*, *z*.
